# The Ins and Outs of Anthrax Toxin

**DOI:** 10.3390/toxins8030069

**Published:** 2016-03-10

**Authors:** Sarah Friebe, F. Gisou van der Goot, Jérôme Bürgi

**Affiliations:** Faculty of Life Sciences, Global Health Institute, Ecole Polytechnique Fédérale de Lausanne, Lausanne 1015, Switzerland; sarah.friebe@epfl.ch (S.F.); jerome.burgi@epfl.ch (J.B.)

**Keywords:** anthrax toxin, endocytosis, protein structure, anthrax toxin receptors, cancer treatment

## Abstract

Anthrax is a severe, although rather rare, infectious disease that is caused by the Gram-positive, spore-forming bacterium *Bacillus anthracis*. The infectious form is the spore and the major virulence factors of the bacterium are its poly-γ-D-glutamic acid capsule and the tripartite anthrax toxin. The discovery of the anthrax toxin receptors in the early 2000s has allowed in-depth studies on the mechanisms of anthrax toxin cellular entry and translocation from the endocytic compartment to the cytoplasm. The toxin generally hijacks the endocytic pathway of CMG2 and TEM8, the two anthrax toxin receptors, in order to reach the endosomes. From there, the pore-forming subunit of the toxin inserts into endosomal membranes and enables translocation of the two catalytic subunits. Insertion of the pore-forming unit preferentially occurs in intraluminal vesicles rather than the limiting membrane of the endosome, leading to the translocation of the enzymatic subunits in the lumen of these vesicles. This has important consequences that will be discussed. Ultimately, the toxins reach the cytosol where they act on their respective targets. Target modification has severe consequences on cell behavior, in particular on cells of the immune system, allowing the spread of the bacterium, in severe cases leading to host death. Here we will review the literature on anthrax disease with a focus on the structure of the toxin, how it enters cells and its immunological effects.

## 1. Introduction

*Bacillus anthracis* is the causative agent of anthrax, which is mainly a disease of herbivores. In general, humans naturally contract the disease when in contact with infected animals or animal products. Before the development of a vaccine in 1937 by Max Sterne, anthrax was the main cause of mortality in cattle, pigs, horses, goats and sheep [1]. Anthrax outbreaks are linked to environmental changes, with heavy rain and temperature being two important factors [1].

Anthrax can be contracted by humans in four different ways: through the skin, by injection, by inhalation and by ingestion. Cutaneous anthrax is the least severe [2] and most common form of the disease, and it can be contracted by spores entering the body through a skin lesion. This form has the lowest mortality rate, less than 1% when treated with antibiotics, and represents 95% of the cases [1]. Inhalational and gastrointestinal anthrax are difficult to diagnose and are associated with high mortality [1]. The first case of anthrax infection by injection was described in 2000, with a more severe outbreak in 2009 [3,4]. It constitutes a new form of anthrax infection that mainly affects soft tissue, and patients require antibiotic treatment as well as surgery to remove the necrotic tissues [5,6]. Even if the decline of anthrax infection in animals and humans has been significant during the last century, the multiple outbreaks among drug-injection users and the 2001 terrorist attacks clearly show that *B. anthracis* infections are still a risk to consider.

The *B. anthracis* life cycle comprises two forms, a dividing vegetative form and dormant spores. Sporulation of *B. anthracis* is a defense mechanism that occurs when the environment cannot sustain the pathogen’s growth, usually when the bacterium is released into the soil after death of the host. *B. subtilis* and *B. anthracis* share a significant portion of spore proteins [7] and spore morphogenesis is conserved between the two species [8]. The spore is formed of several layers, the core containing the genetic material decorated by protective small acid-soluble proteins [9] surrounded by an inner cortex that will be the precursor of the cell wall of the vegetative state. A thick proteinaceous coat surrounds the cortex and protects the inner core, preventing dehydration. The spores are extremely resistant and, given the right environmental condition, can survive up to 200 ± 50 years [10]. Finally, spores are the infectious agents leading to anthrax infection.

The vegetative form of *B. anthracis* produces several virulence factors in the form of exotoxins and a capsule. These virulence factors are mainly encoded by two virulence plasmids called pXO1, responsible for the bacterium exotoxins, and pXO2 that encodes for the biosynthetic machinery responsible for the production of the capsule. The bacterium’s capsule is composed of poly-γ-D-glutamic acid (PGDG), a linear polymer of low immunogenicity [11], and has been shown to confer resistance against phagocytosis and the complement system [12,13].

Here we will review recent findings that highlight how anthrax toxin hijacks different cellular mechanisms to increase its intoxication efficiency and describe new structural data that explain the molecular mechanisms responsible for the almost unmatched efficiency of this toxin to disrupt cellular functions and kill its host.

## 2. Anthrax Toxin Receptors and Toxin Endocytosis

### 2.1. Cellular Entry and Endocytosis

Once the spores have germinated in the body of the infected host, the bacteria will produce anthrax toxin. This tripartite AB toxin is composed of the receptor-binding subunit, protective antigen (PA), and the two enzymatic subunits, lethal and edema factor (LF and EF). Together, these subunits can form two active toxins, lethal toxin (PA + LF) and edema toxin (PA + EF). To affect the cells of the host, the toxins needs to gain access to the cell cytoplasm where the two enzymatic subunits of the toxin act. To do so, anthrax toxin has hijacked cellular pathways, a common strategy of pathogens. In the case of anthrax toxin, there are two main receptors: tumor endothelial marker 8 (TEM8, ANTXR1) and capillary morphogenesis gene 2 (CMG2, ANTXR2), which were discovered in 2001 and 2003, respectively [14,15]. The physiological role of these two highly homologous receptors is poorly understood. Most of the results obtained to date point to an involvement of the receptors in the homeostasis of the extracellular matrix [16]. Given their similarity to integrins, they seem to bind to proteins of the extracellular matrix (ECM), such as collagens and fibronectin [17,18,19] and might regulate the accumulation of these *in vivo* [20,21,22]. Other cell surface proteins have been described as receptors, such as β1-integrin [23] or the Wnt signaling co-receptor LRP6 [24,25]. Given that mice are completely resistant to anthrax toxin challenge when CMG2 is knocked out [26], the proteins described above might be only accessory, rather modulating than mediating entry.

Both CMG2 and TEM8 are type I transmembrane proteins, with an extracellular van Willebrand factor A (vWA) domain, an Ig-like domain, a single transmembrane helix and a cytoplasmic tail [16]. The vWA domain is involved in ligand binding and does so via its metal-ion-dependent adhesion site (MIDAS) domain [15]. Interestingly, although the vWA domains of CMG2 and TEM8 are highly conserved, the affinity of PA for CMG2 is higher, ranging from 10- (cell culture) to 1000-fold (*in vitro*) [27,28]. This could partly explain why CMG2 is the main toxin receptor in mice [27].

**Figure 1:**
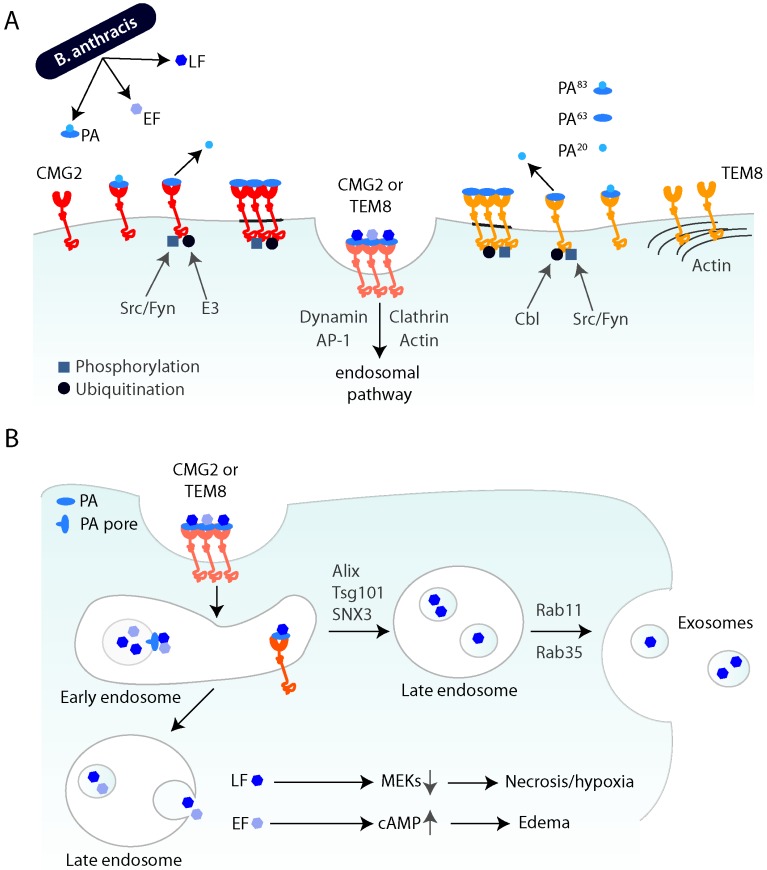
Schematic overview of cellular entry of anthrax toxin and progression through the endocytic pathway (**A**) *B. anthracis* produces the three subunits of anthrax toxin: protective antigen (PA), lethal factor (LF) and edema factor (EF). PA^83^ binds to either CMG2 or TEM8 at the cell surface, where it is cleaved by furin, leading to the receptor-associated PA^63^ and soluble PA^20^. Upon toxin binding, the receptors are phosphorylated by Src-like kinases, namely Src or Fyn, and are ubiquitinated by Cbl (for TEM8) or an unknown E3 ligase (for CMG2). After oligomerization in lipid rafts (dark grey), the receptor-toxin complex is internalized by endocytosis. This endocytosis seems to largely depend on clathrin, dynamin, AP-1 and actin. (**B**) After trafficking to the early endosome, PA can undergo a conformational change, leading to pore formation and translocation of the enzymatic subunits across the membrane. LF and EF get to the cytoplasm by either direct translocation or by backfusion of intraluminal vesicles (ILVs) of late endosomes. LF cleaves MEKs and thereby leads to necrosis and hypoxia. EF leads to the elevation of intracellular cAMP and causes edema. LF can also take an alternate route and be packaged into ILVs, which are then released as exosomes and can transmit the toxin to naïve cells.

After binding to the receptor at the cell surface, the 83 kDa form of PA (PA^83^) is cleaved by furin-like proteases and produces a shorter form, PA^63^ [29,30]. Only this cleaved form of PA is able to assemble into oligomers, either heptamers or octamers [31,32]. The receptor-toxin oligomers cluster in lipid rafts, which are subdomains of the plasma membrane [33]. The localization of the oligomers to these subdomains is important, as disruption of lipid rafts leads to a defect in subsequent internalization of the toxin [33]. Partitioning into lipid rafts is controlled by palmitoylation of the tail of TEM8. Palmitoylation keeps the receptor out of lipid rafts and palmitoylation-deficient TEM8 will rapidly internalize, thereby perturbing the finely tuned timing of this process [34]. This timing and correct functioning of these processes is also mediated by other posttranslational modifications of the receptor tail. Both receptors are phosphorylated on tyrosine residues by Src or Fyn in response to PA binding. This is not crucial for formation of the oligomers but impairs toxin entry [35]. Another important modification is ubiquitination. After toxin binding, β-Arrestin2, a well-known adaptor protein in endocytosis [36], binds to the tail. This leads to the recruitment of an E3 ubiquitin enzyme and the toxin-induced ubiquitination of the two receptors [37]. The E3 ligase of TEM8 is Cbl, while the enzyme ubiquitinating CMG2 has not yet been established [34,37] (Figure 1A).

LF and EF will only bind to PA oligomers, with a 3:1 stoichiometry of enzymatic subunit to heptamer and 4:1 to octamer. The hetero-oligomeric toxin-receptor complex is subsequently internalized, mainly by clathrin-mediated endocytosis, although alternate, context-dependent pathways may exist [37,38]. Interestingly, the clathrin-dependent endocytosis of anthrax toxin seems to depend on the unconventional adaptor AP1 rather than on the more common AP2 [37]. Another important player in enabling entry of the toxin into cells is the actin cytoskeleton. Endocytosis of both receptors after toxin binding depends on actin and TEM8 appears to be pre-organized by actin at the cell surface (Figure 1A) [37]. It has also been proposed that toxin entry depends on the reorganization of the actin cytoskeleton by calpain [39]. Finally, a genome-wide screen for anthrax susceptibility genes led to the identification of ARAP3, a protein involved in Rho-mediated actin remodeling [40], further linking actin to toxin entry.

Once internalized from the cell surface, PA undergoes a conformational change triggered by the low pH in endosomes and forms a pore that acts as a translocation channel for the enzymatic subunits. The precise site along the endocytic pathway where pore formation occurs may depend on whether entry is mediated by TEM8 or CMG2. If PA is bound to TEM8, dissociation and pore formation occurs at earlier steps in the endocytic pathway, presumably in early endosomes, whereas for CMG2, the pH threshold is lower and seems to require trafficking to late endosomes [41]. To get to the cytoplasm, EF and LF translocate through the PA pore, either directly to the cytoplasm [42] or first by translocation into intraluminal vesicles (ILV) and then backfusion of these with the limiting membrane of endosomes [43]. The latter, more complex pathway, offers two major advantages. First, the enzymatic subunits can remain in the lumen of ILV protected from lysosomal enzymes, allowing long-term action of the toxin, as observed *in vivo*. Also, by residing in ILVs, the toxin can be released to the extracellular environment as exosomes and taken up into cells independently of the receptor and invisible to the immune system [44]. The loading of LF into these exosomes depends on Alix and Tsg101, components of the ESCRT complexes and SNX3, a sorting nexin involved in the retromer complex. Release of the LF-exosomes then depends on the action of the two small GTPases Rab11 and Rab35 (Figure 1B) [44]. This entire mechanism allows both long-term storage and long-range transmission of the toxin. The long-term and very potent activity of the anthrax toxin might explain why patients, even without detectable bacterial load after successful antibiotic treatment, still succumb to the disease.

Apart from entry via the plasma membrane, the enzymatic subunits LF and EF might also reach the cytoplasm via a different pathway. In a scenario described by Banks *et al.*, spores are phagocytosed by macrophages, germinate in the phagolysosome, and produce the anthrax toxin there. Retrotranslocation is achieved by formation of the PA pore directly in the limiting membrane and depends on the presence of the anthrax toxin receptor [45].

### 2.2. Cellular Effects of Anthrax Toxin

*B. anthracis*, apart from the anthrax toxin, produces other virulence factors. The poly-d-glutamic acid capsule is an excellent defense of the bacteria against phagocytosis and is an important determinant of establishing successful infection *in vitro* and *in vivo* [12,46]. More factors have been postulated but remain to be tested for their exact role in anthrax. The anthrax toxin, together with the capsule, however, seem to remain the two most important virulence factors.

Lethal factor (LF) is a zinc-dependent metalloprotease, which cleaves members of the MAP kinase kinase family (MEK), leading to their inactivation [47]. Downstream of MEKs lie the ERK, JNK and p38 pathways, which are important regulatory pathways of cell stress, growth and other stimuli. Edema factor (EF) is a highly efficient calmodulin-dependent adenylyl cyclase. It can convert up to 2000 molecules of ATP to cAMP per second, significantly increasing the concentration of this second messenger [48]. Both rely exclusively on PA to get into cells.

Anthrax toxin acts both at early and late stages of infection and both EF and LF seem to have preferential target sites and complementary roles to ensure successful progression of the disease. After entering the host and germination, the first mission for anthrax toxin is to incapacitate the immune response. It seems to first target myeloid cells, such as macrophages and neutrophils [49], paving the way for the bacterial replication. Mice that were specifically deleted for CMG2 in their myeloid lineage were resistant to anthrax infection, underlining the importance of these immune cells in the establishment of a successful infection [49]. The primary effector here seems to be LF, which inactivates MEKs, thereby promoting apoptosis of the cells [50,51,52]. In addition to targeting MEKs, LF can have another cellular target. Some mice strains and rats harbor a polymorphism in NLRP1, a component of the NLRP1 inflammasome, which makes it susceptible to cleavage by LF, thereby leading to a rapid, caspase-1-dependent apoptosis [53]. Interestingly, having susceptible macrophages seems to protect these mice more from infection than having resistant macrophages [54,55]. This seemingly counterintuitive result can be explained by an elevated secretion of inflammatory cytokines by susceptible macrophages, thereby leading to an activation of the immune response, which then efficiently controls the spread of the bacteria.

Therefore, LF-induced apoptosis has to be a rather slow process, which is also thought to help bacteria reach lymph nodes, a key environment for replication and dissemination [56,57,58]. After *B. anthracis* is phagocytosed by macrophages or dendritic cells [59,60], the anthrax toxins enable a “Trojan horse” delivery of the pathogen to the lymph nodes [61]. To achieve this, EF adenylate cyclase activity is believed to delay the LF-mediated macrophage apoptosis. Indeed, the high cAMP levels created by EF activate the transcription factor CREB via PKA and this seems to induce transcription of PAI-2, a regulator of cell survival [62]. This counteracts the pro-apoptotic cleavage of MEK by LF, resulting in a balance between survival and programmed cell death. This balance is finely tuned to ensure maximum infection efficiency and dissemination of the pathogen to the lymph nodes [62]. In addition to this, EF can upregulate the expression of CMG2 via the transcriptional regulator CREB, increasing susceptibility of the cells to the toxin [63,64] and can delay general protein clearance in serum *in vivo* by a still unknown mechanism, thereby prolonging toxin effects in circulation [65].

Other effects of EF have been described but are less clear. Some studies show EF as an effector in increasing motility of macrophages, helping in dissemination of the bacteria [66,67], whereas others claim it blocks motility and migration of immune cells, impairing their arrival at inflammatory sites [68,69].

What is known about how LF and EF act during infection is dependent on several parameters such as the animal model, the entry route of the spores and/or the toxins and their respective dosage. In some cases, infection depends largely on the presence of the toxins, whereas in others, pathogenicity can be more toxin independent [70,71]. Also, the relative contribution of LF and EF to disease progression can vary. LF has been repeatedly described as more important to sustain infection in different animal models [72,73,74], yet other reports also highlight the importance of EF at different stages of infection [66,75,76]. However, the consensus view is that both anthrax toxins contribute to pathogenicity and fatality of anthrax infections by disabling the immune system and by damaging vital functions of the host.

More specifically, both LF and EF target and block the immune response in an early stage of systemic infection, allowing the infection to progress to later stages. The first stage of infection can be asymptomatic, followed by an acute stage, with rather non-specific symptoms, such as fever, sore throat, vomiting and diarrhea for inhalational and gastrointestinal anthrax [77]. The cause of death is a combination of bacterial sepsis, as bacteria rapidly multiply, and also toxemia, caused by a high level of anthrax toxin in circulation, affecting multiple organs [78]. That toxins are important for these late stages becomes clear with the fact that even patients with no detectable bacterial load after antibiotic treatment can die from a systemic infection. This is probably due to a prolonged action of the toxins in circulation [44].

As CMG2 is a rather ubiquitiously expressed receptor, toxins could potentially affect a large variety of tissues in the host. However, both LF and EF seem to have a preference for certain tissues: LF targets cardiomyocytes and smooth muscle cells, thereby affecting the cardiovascular system, whereas EF primarily targets hepatocytes, damaging the liver [79]. Mice deleted of CMG2 in cardiomyocytes and smooth muscle cells are fully resistant to LF. However, only about 80% of the hepatocyte-specific knockout mice of CMG2 are resistant to EF. This indicates that LF targets primarily the heart, but EF has other tissue targets apart from the liver [79]. EF, as the name implies, causes edema in skin and liver, though the exact mechanism remains unclear.

## 3. Toxin Structure

### 3.1. Protective Antigen

**Figure 2:**
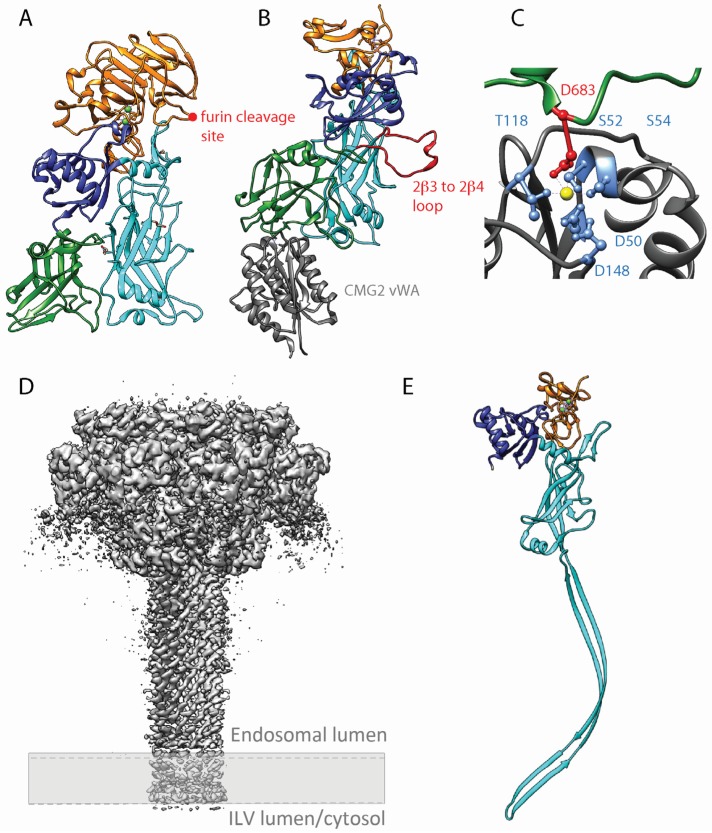
Protective Antigen structure in monomeric and oligomeric form. (**A**) X-ray structure of a PA monomer deleted for the membrane insertion loop (based on the PDB 3TEW structure). The deletion of this very flexible loop usually absent from previous crystal structures allowed a significant increase of the structure resolution [89]. Domain 1 is represented in orange, domain 2 in cyan, domain 3 in blue and domain 4 in green. The furin cleavage site is indicated with a red dot. (**B**) X-ray crystal structure of PA bound to CMG2 vWA domain (based on PDB 1TZN) [83]. Domain 1′ (cleaved domain 1) is represented in orange, domain 2 in cyan, domain 3 in blue and domain 4 in green and CMG2 vWA domain in dark grey. The 2β3–2β4 membrane insertion loop is colored in red. (**C**) Detail of CMG2 MIDAS interacting with PA Asp683 (based on PDB 1TZN). The residues involved in the metal ion coordination are colored in light blue and the PA Asp bound to the metal ion in red. (**D**) PA pore structure by Cryo Electron microscopy. The cryoEM map view from the side has a resolution of 2.9 Å (based on PDBE EMD-6224). The approximate position of the membrane is depicted with a discontinuous line and grey rectangle. (**E**) Structure of the PA monomer after pore formation and membrane insertion based on *de novo* atomic modelling (based on PDB 3J9C) [81]. Cleaved domain 1′ is represented in orange, domain 2 in cyan, domain 3 in blue. Due to its flexibility in the structure the domain 4 is not represented.

Protective antigen is the non-catalytic subunit of the anthrax toxin that binds to CMG2 and TEM8 and triggers toxin uptake [33]. PA is composed of four domains [80]. Domain 1 (residues 1 to 258) is cleaved by furin [30] after a RKKR^196^ motif localized on an solvent-exposed loop (Figure 2A). This cleavage is a prerequisite for PA oligomerization. Domain 2 (residues 259 to 487) has a modified greek-key topology and a large flexible loop between strands 2β2 and 2β3, known to be implicated in membrane insertion (Figure 2B in red) [81]. Domain 3 (residues 488 to 595) resembles domain A of toxic-shock-syndrome toxin 1 [82]. Finally, domain 4 (596–735) forms a β sandwich with immunoglobulin-like fold (Figure 2A). This domain is strongly involved in receptor binding (Figure 2B). Indeed, the aspartic acid 683 has been shown to bind to the magnesium ion complex by the receptor MIDAS motif (Figure 2C) [83].

There are multiple histidines in the greek-key motif of domain 2 that are potentially titrated during pH change in the endosomes. Protonation of these residues was postulated to lead to a change in conformation in the 2β2 and 2β3 strands of domain 2 leading to membrane insertion after acidification. However, PA labelled with fluoro-histidine, residues that are resistant to protonation, was still able to form a pore. Interestingly, the pore formed by the modified PA was unable to translocate LF, which indicated that some histidine residues play a role in this mechanism [84]. In addition, the loop connecting domain 2β3 to 2β4 was shown to undergo an order-to-disorder transition when the pH of the solution in which the crystals were grown was decreased from 7.5 to 6. This could be the molecular changes that lead from pre-pore to pore formation and membrane insertion [80].

### 3.2. Binding of PA to Its Cellular Receptor

PA interacts with the vWA domain of the receptors through domains II and IV (Figure 2B). Apart from the Asp683 interacting with the metal ion coordinated by the MIDAS, CMG2 α2–α3 loop binds to a deep groove formed by an edge of domain 4 β sandwich. This edge in groove interaction seems to be conserved in TEM8. In addition, the 2β3–2β4 loop of PA domain 2 inserts in a pocket in CMG2, which further stabilizes the interaction (Figure 2B). Interestingly, this part of PA is the one that inserts into the endosomal membrane after conversion from pre-pore to pore. Thus, it is believed that the anthrax receptor stabilizes the pre-pore structure and maintains specific constrains that block spontaneous conversions [83].

### 3.3. PA Pore Structure

The *in vitro* pre-pore to pore conversion usually leads to rapid aggregation of the proteins. However, recently a high resolution EM structure of the PA pore was obtained by directly converting the pre-pore into the pore by acidic pH on electron microscopy grids. This method generated a complete structure of the anthrax toxin pore at an overall resolution of 2.9 Å (Figure 2D) [81]. The PA pore formed a 14-stranded β-barrel structure similar to the α-hemolysin pore from *Staphylococcal aureus* [85] and the cytolysin from *Vibrio cholera* [86]. The pore opening had a radius varying from 16 Å to 10 Å that can only accommodate secondary structures such as alpha helices. Roughly 40 Å after the beginning of the PA pore, the seven F427 from each monomer pointing their side chains into the pore lumen form a 6 Å ring called the Φ clamp. The aromatic plane is parallel to the channel central axis and it has been shown that this clamp is required for LF and EF proteins translocation through non-specific hydrophobic interactions. As this structure is smaller than the secondary structures of LF and EF, the translocating proteins are required to be completely unfolded to go through the channel.

To facilitate protein translocation after unfolding, the PA pore appears to reduce the energy penalty caused by exposure of hydrophobic patches by binding and stabilizing them with the Φ clamp [87]. In addition, the Φ clamp is thought to maintain the proton gradient measured between the endosomal compartment and the cytosol/ILV lumen [88].

Jiang and colleagues observed that multiple conformational changes in domain 2 are responsible for the pre-pore to pore conversion. In their study of the structure of the PA pore, they described a tree layer mechanism, with first a change in conformation of the loop β10–β11 of domain 2 due to acidification. Second, an ordering of the domain 2 β7–β8, β5–β6 and β11–β12 loops leads to the formation of the Φ clamp and finally the separation of domain 2 with domain 4 leads to the refolding of the membrane insertion loop into the endosomal membrane [81].

### 3.4. PA Oligomer Diversity

In a recent study, the position of domain 4 of PA was shown to be one of the structural determinants of PA oligomeric stoichiometry [89]. Depending on this domain orientation according to domain 2, a PA_8_ or PA_7_ stoichiometry can be favored. Indeed, PA oligomers exist in two forms, a main heptameric complex and an octameric oligomer that represents around 30% of the total population. The PA pre-pore assembly through dimeric intermediates either caused by receptor dimers or LF/EF mediated dimerization is believed to be cause of octameric complexes [31]. In addition, the octameric PA pre-pore was found to be more stable in blood serum than its heptameric counterpart. Indeed, it was observed that a transition from pre-pore to pore can happen *in vitro* under physiological conditions for PA heptamers. Yet, in the absence of membrane this transition can lead to protein aggregation. In line with their increased stability, PA octamers retain their lipid bilayer insertion capacity for a longer time than PA heptamers, thus they are able to lyse macrophage for a longer period [90]. This mixed population of PA oligomers is postulated to be important to retain toxicity in all physiological conditions encountered by the bacteria. As PA octameric complexes are stable at fever-like temperatures while the heptameric ones are not, the toxins are still able to exert their action when the host immune system reacts to the *B. anthracis* infection.

### 3.5. Lethal Factor Structure

LF is a 90 kDa zinc metalloproteinase and together with Edema Factor constitutes the catalytic subunits of the anthrax toxin. In combination with PA it forms the Lethal Toxin, known to cleave with a very high specificity the MAPKK (MEK) family of proteins [47]. Its crystal structure was resolved in 2001 and showed that the protein is composed of four domains. The catalytic activity is carried out by domain 4, with a HEFGF motif that coordinates a Zn^2+^ ion. Domain 1 is structurally similar to domain 4, without the Zn^2+^ coordination site and mediates the interaction with PA. This domain only slightly contacts domain 2 and has been used to allow heterologous proteins, such as chimeric fusion proteins to bind and translocate through the PA pore. An example for this is FP59, a fusion of residues 1–254 from LF and the catalytic domain of *Pseudomonas* exotoxin A [91].

### 3.6. Edema Factor Structure

Edema factor is a 92.5 kDa adenylate cyclase known to quickly and efficiently increase cyclic AMP levels in eukaryotic cells [92]. The protein is activated by calmodulin, a protein present in host cells, and is 1000 more efficient than mammalian adenylyl cyclase [93]. The catalytic domain of EF is composed of three globular domains, with the active site being at the interface of two domains. Binding to calmodulin induces global changes in EF structure, with the most drastic changes observed in the N-terminal helical domain. This structural rearrangement is responsible for EF activation and production of cAMP [94].

### 3.7. LF/EF Translocation

Translocation of LF and EF from the endosomal compartment to the cytosol is a process that does not require any energy. Differences in proton concentration and electrical potential appear to be the only requirements for efficient transport across the PA pore [88,95,96]. However, protein translocation requires the unfolding of both LF and EF, as the pore diameter is too narrow (6 Å at the minimum) to accommodate a folded protein.

LF translocation occurs from the N-terminus of the protein, with LF α1β1 unfolding upon PA binding and inserting in a cleft formed by adjacent PA subunits [97]. In addition to the PA pore formation, the low pH found in early and late endosomes is destabilizing EF and LF conformations, a mechanism that will allow for protein unfolding and rapid translocation [98]. Interestingly, *in vitro* experiments showed that the ΔpH between the *cis*, *i.e.*, the initial compartment, and the *trans*, *i.e.*, the receiving compartment, was promoting the translocation of LF even if the membrane potential is low (+20 mV) [88]. Thus, the toxins were designed to use the low endosomal pH to unfold and the ΔpH to travel through the PA pore and reach the cytoplasm.

In accordance, in the cellular environment, drugs like Bafilomycin A1 that block the vacuolar ATPase proton pump and thus endosome acidification [99] or neutralize their acidic environment such as chloroquine [100] are known to inhibit LF/EF translocation to the cytosol [101,102].

Both toxins are thus thought to follow a charge-state Brownian ratchet translocation model, where the Brownian motion of the unfolded toxins is oriented with the help of a chemical gradient and the Φ clamp. This structure binds and stabilizes hydrophobic sequences in the unfolded EF/LF and directs the proteins translocation across the PA pore according to the ΔpH [88].

## 4. Conclusions and Outlook

*Bacillus anthracis* is a well-adapted and very efficient pathogen of the *Bacillus* genus [103]. The major virulence factor, anthrax toxin, contributes significantly to the pathogenic success of *B. anthracis*. It is both capable of efficiently hijacking the endocytic pathway and shielding itself from degradation in intraluminal vesicles. This allows a sustained intoxication of cells that can last for days in addition to toxin transmission through the exosomal pathway that can infect cells devoid of anthrax toxin receptors [44]. The catalytic subunits LF and EF are highly efficient enzymes; minute amounts of each protein are enough to severely perturb cellular function. Anthrax toxins were also selected for fast and resilient cytoplasmic translocation. For this reason, PA has been a prime target for engineers to deliver polypeptides and compounds to the cell cytoplasm.

In a recent study, PA was used as a tool to deliver antisense oligonucleotides and siRNA to the cytoplasm of eukaryotic cells. The method seems to be efficient and of low toxicity. Coupling this delivery system with the targeting properties of engineered PA could be of great use in *in vivo* downregulation of target genes [104]. Another study showed that PA was also capable of translocating non-canonical polypeptides, nicely illustrating the broad translocation ability of the PA pore [105].

PA has also been used as a novel drug in targeting cancer cells. One approach involves the targeting of PA to cells overexpressing TEM8. This receptor has been shown to be upregulated during tumor angiogenesis and provides a convenient target for anti-angiogenic therapy [106,107]. As TEM8 is a quite broadly expressed protein, this approach had to be slightly modified to reduce toxic side effects. The concept of PA as a vehicle to transport cytotoxins to specific cells was first experimentally verified in 2000 [108]. To become active and form an endocytosis-competent oligomer, PA needs to be specifically cleaved by furin, as stated previously. Leppla and coworkers re-engineered a PA cleavage site to become specific to metalloproteinases, which are known to be significantly upregulated in multiple cancer cell lines [109]. A combination of the mutant PA and FP59 was then used to test the tumor-cell specificity of the construct. The PA + FP59 combination specifically targeted and killed tumor cell lines, but was not toxic to non-tumoral bystander Vero cells [108]. The same strategy can be applied to toxins engineered to be cleaved by urokinase. The urokinase plasminogen activator (uPA) system appears to play a crucial role in multiple cancers [110] and a study showed that an engineered PA cleaved by activated urokinase in combination with FP59 had a significant anti-tumoral activity in mice transplanted with tumors of different origin [111].

There is a certain drawback to these approaches. Although efficient, these toxins retain some unspecific toxicity *in vivo.* A recent study tested mice survival and *in vivo* tumoricidal activity of these engineered constructs. The gastrointestinal tract was the most damaged tissue after intravenous or intraperitoneal administration of the toxins. This off-target effect is believed to be the result of high MMP activity due to regular tissue repair. A construct where the simultaneous presence of MMPs and uPA is required for activation seems to be the most promising approach, as it retains efficiency but has reduced unspecific toxicity [112].

Anthrax, the anthrax toxin and the cellular anthrax toxin receptors have been subject to intense research over the last few decades. Apart from fascinating insights into the pathological functioning of the toxin both *in vitro* and *in vivo*, the work on the anthrax toxin and receptors has provided us with a better understanding of cellular mechanisms, such as endosomal dynamics. The most recent research. Moreover, clearly indicates the highly intriguing possibility of hijacking and manipulating anthrax toxin for use in cancer research.

## Abbreviations

The following abbreviations are used in this manuscript:

PA	Protective Antigen
LF	Lethal Factor
EF	Edema Factor
ANTXR1	Anthrax toxin receptor 1
ANTXR2	Anthrax toxin receptor 2
